# Mastering Transabdominal Preperitoneal (TAPP): A 5,124 Case Journey in Hernia Repair With a Singular Approach and Our Results

**DOI:** 10.7759/cureus.104758

**Published:** 2026-03-06

**Authors:** Mohit Bhatia, Sharmila Vijayan, Doaa Al-Maliki, Shamsi El-Hasanii

**Affiliations:** 1 General Surgery, Princess Royal University Hospital, Orpington, King's College, London, GBR; 2 Upper GI Surgery, Princess Royal University Hospital, Orpington, King's College, London, GBR

**Keywords:** abdomen ventral hernia, inguinal hernia repair, laparoscopic inguinal hernia, tapp repair, transabdominal preperitoneal (tapp)

## Abstract

Background

Inguinal hernia is a common surgical condition resulting from a defect in the myofascial plane, with both congenital and acquired etiologies. Although open repair has traditionally been the standard approach, over the last decade, laparoscopic repair, particularly the transabdominal preperitoneal (TAPP) method, has gained popularity due to its diagnostic and therapeutic advantages. However, its adoption has been limited due to technical complexity and perceived complication risks.

Objective

To present our technique, perioperative strategy, and outcomes in a large cohort of patients undergoing laparoscopic TAPP hernia repair, with a focus on refining surgical steps to optimize safety and efficacy.

Methods

A retrospective review was conducted on laparoscopic TAPP hernia repairs performed on 5,124 patients over a defined period. Institutional ethical approval was obtained. Preoperative assessments included dynamic imaging and individual risk stratification. Surgical technique emphasized wide preperitoneal dissection, tailored mesh placement with at least 3 cm overlap, and meticulous closure of peritoneal flaps. A four-port approach was used, and a variety of groin hernias (inguinal, femoral, and obturator) were included. Concurrent umbilical and Spigelian hernias were repaired when encountered.

Results

Of the 5,124 patients, 4,252 were males (83%), and 872 were females (17%). The technique proved feasible across a broad body mass index (BMI) spectrum (19-46) and in patients with prior abdominal surgery. Complications were minimal, conversion to open surgery occurred in 13 patients (0.25%) of cases, and recurrence rates were low (less than 0.3%). 153 patients (3%) developed seroma/hematoma formation, whereas 256 patients (5%) developed urinary retention postoperatively. The tailored use of overlapping mesh segments and cautious dissection minimized the risk of recurrence and postoperative complications such as seroma or chronic pain.

Conclusion

Our experience with laparoscopic TAPP repair suggests that, when performed by an experienced surgeon using a standardized technique and with careful attention to anatomical detail, this approach can be safe and effective. When utilised by an experienced surgeon, TAPP enables comprehensive groin assessment, supports early recovery, and can deliver durable repair outcomes, including in more complex cases. With appropriate training, these findings support the continued and considered adoption of the TAPP approach in hernia surgery.

## Introduction

An inguinal hernia develops when there is a defect in the myofascial layer formed by the internal oblique and transversus abdominis muscles, which allows part of the abdominal contents to protrude through the defect. Inguinal hernias can be either acquired or congenital, with the majority being acquired. Conditions that increase intra-abdominal pressure, as well as connective tissue disorders, can also contribute to the development of inguinal hernias [1]. Congenital hernias result from a patent processus vaginalis, while acquired inguinal hernias are thought to occur due to an imbalance between type III and type I collagen [2].

Inguinal hernias make up approximately 75% of all abdominal wall hernias. For many years, open repair has been the standard treatment [3]. However, over the past 10-15 years, the laparoscopic approach has become increasingly popular with both surgeons and patients. There has been an ongoing debate about which method provides the greatest benefits. Among laparoscopic techniques, both total extraperitoneal repair (TEP) and trans-abdominal preperitoneal repair (TAPP) are commonly used, but there is no clear consensus on which is superior. Some evidence suggests that TEP may have a higher overall complication rate but a lower risk of intra‑abdominal injury, whereas TAPP may be associated with lower recurrence rates [4].

Unlike other laparoscopic procedures, like cholecystectomy, laparoscopic inguinal hernia repair has not been widely adopted. This may be attributed to factors such as a steep learning curve, limited training opportunities, and limited public awareness [5].

Inguinal hernias are common conditions that usually require surgical intervention. The two primary surgical approaches-open repair and laparoscopic repair-each have distinct advantages and considerations. The comparative outcomes depend on several factors, including recurrence rates, postoperative pain, operative time, and complication rates [6].

This series focuses on the repair of groin hernias, including inguinal hernias (both direct and indirect), femoral hernias, and obturator hernias. Over the years, our approach to preoperative, operative, and postoperative care for groin hernia repair has evolved as we have gained more experience with this common pathology in our surgical practice.

## Materials and methods

Methods 

In our retrospective cohort, patients aged 18 years and above were included, with no upper age or body mass index (BMI) limit. The oldest patient in the series was 91 years old. Previous abdominal or pelvic surgery was not considered a contraindication to laparoscopic repair. Patients with a history of radical prostatectomy were excluded from the laparoscopic approach and were advised to undergo open groin hernia repair.

In patients aged over 60 years, the contralateral groin was routinely assessed for occult hernias due to the high prevalence observed in this age group. When a contralateral hernia was suspected clinically, confirmation was obtained using dynamic groin ultrasonography before discussion of operative management. This approach resulted in an increased proportion of laparoscopic bilateral inguinal hernia repairs. Patients presenting with obstructed or strangulated groin hernias were managed laparoscopically whenever feasible.

For patients with atypical chronic groin pain, preoperative evaluation included groin ultrasonography and pelvic magnetic resonance imaging. Orthopaedic consultation was obtained when indicated. Patients were counselled preoperatively that symptomatic improvement was anticipated but could not be guaranteed.

Patients with large inguinoscrotal hernias, particularly those that were clinically reducible, were included. In most cases, hernia contents could be reduced under general anaesthesia, allowing a laparoscopic approach; conversion to open repair was undertaken when reduction was not possible.

All patients underwent preoperative anaesthetic assessment, received written information, and attended a dedicated counselling session addressing the operative procedure and postoperative expectations. This included advice on early mobilisation, regular analgesia, the risk of lateral thigh paraesthesia, and the potential for postoperative seroma formation.

Operative technique

All procedures were performed under general anaesthesia with the patient in the supine position. Pneumoperitoneum was established using a Veress needle, initially at an operating pressure of 20 mmHg, which was gradually reduced to 15 mmHg during the procedure to minimize surgical emphysema. Routine urinary catheterization was performed in patients over 60 years of age and in those undergoing bilateral inguinal hernia repair. The catheter was removed a few hours postoperatively once the patient was able to mobilize.

Port placement consisted of a 5 mm port inserted 1 cm above the umbilicus, with additional right and left 5 mm ports placed at the lateral borders of the rectus abdominis at the level of the umbilicus. A 10 mm port was positioned laterally on the contralateral side of the hernia repair, just above the anterior superior iliac spine. In bilateral hernia cases, the 10 mm port was placed on the side of the smaller hernia. This port was used for mesh insertion and the introduction of Vicryl sutures.

This port placement strategy was adopted to reduce the risk of port-site incisional hernia. Following desufflation, the muscular defect at the lateral 10 mm port site is protected by the iliac bone, unlike umbilical placement, which carries a higher risk of incisional herniation.

A transverse peritoneal incision was made approximately 2 cm above the apex of the hernia, extending from the medial umbilical ligament laterally as required to allow wide dissection from the midline to the anterior superior iliac spine. Upper and lower peritoneal flaps were carefully dissected using a diathermy hook, guided by key anatomical landmarks including the inferior epigastric vessels, pubic bone, vas deferens and spermatic cord in men, and the round ligament in women. In obese patients, Maryland forceps were commonly used to facilitate preperitoneal dissection.

Wide preperitoneal dissection was performed to identify all potential weak areas, including indirect, direct, femoral, and obturator hernia sites. When encountered, hernia sacs and contents were reduced. In cases where the sac extended deeply into the scrotum and was difficult to dissect safely, the sac was circumcised, and the peritoneal defect was closed using Vicryl sutures.

The primary mesh was tailored to overlap the posterior aspect of the pubic bone medially and to cover all potential weak zones, including the femoral canal, obturator foramen, and inguinal region. A second mesh was added as required to cover the lateral and superior aspects of the inguinal area. Mesh fixation was achieved by using Vicryl suture. Absorbable tackers were reserved for cases with wide defects to prevent mesh migration. Haemostasis was achieved under normotensive anaesthesia. The peritoneal flaps were closed with a continuous Vicryl suture, and any additional peritoneal defects were closed individually. Laparoscopic repair of femoral hernias was found to be generally safer and technically easier compared with the open approach. If necrotic contents were identified, resection was performed either laparoscopically or via a lower midline mini-laparotomy.

Particular care was taken during dissection around the spermatic cord, inferior epigastric vessels, and vas deferens to preserve blood supply and ensure meticulous haemostasis. Injuries to the vas deferens or inferior epigastric vessels were managed by proximal and distal clipping or suturing. In female patients, the round ligament was routinely double-clipped and divided to facilitate elevation of the lower peritoneal flap. Minor spermatic cord injuries were managed with suturing or diathermy; no complete transections were encountered.

The standard approach involved dissection and reduction of the hernia sac. In cases of large inguinoscrotal hernias or significant fibrosis at the neck of the sac, particularly near the inferior epigastric vessels, sac circumcision was performed, leaving sufficient tissue for secure peritoneal closure. For large hernia necks, the use of two overlapping meshes was observed to reduce postoperative seroma formation. Absorbable tackers were routinely used in large defects to prevent mesh displacement.

Patients with previous abdominal surgery were included, and adhesiolysis was generally feasible, allowing successful completion of TAPP repair. In partially reducible hernias, reduction was often achieved following anaesthesia. Gentle laparoscopic traction was always attempted first. In difficult cases, clipping and dividing the inferior epigastric vessels provided improved access to deeply located scrotal contents.

Ports were removed under direct vision to identify and control any bleeding. Skin closure was performed using absorbable sutures. Routine fascial closure for a 5 mm port was not required; however, in selected elderly patients with weakened abdominal wall musculature, 5 mm port fascial defects were closed.

**Figure 1:**
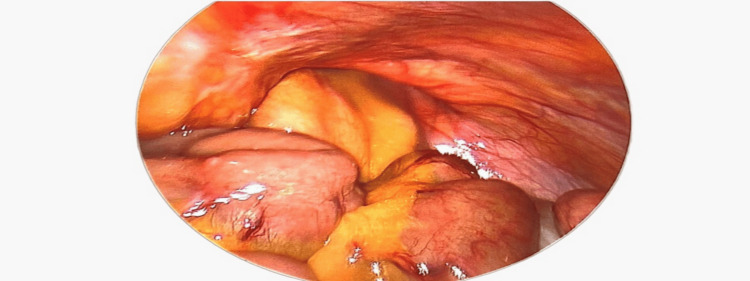
Showing inguinal hernia orifice with contents of the hernia.

**Figure 2:**
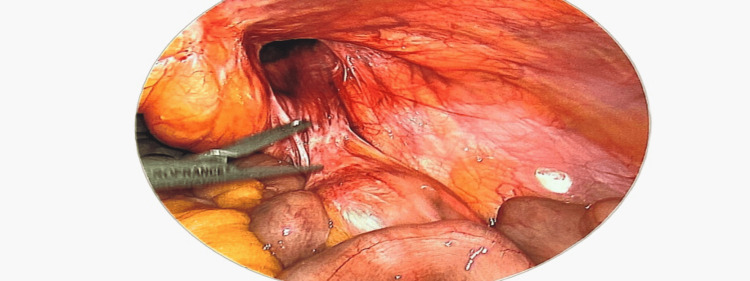
Image demonstrating the appearance after reduction of the hernial contents.

**Figure 3:**
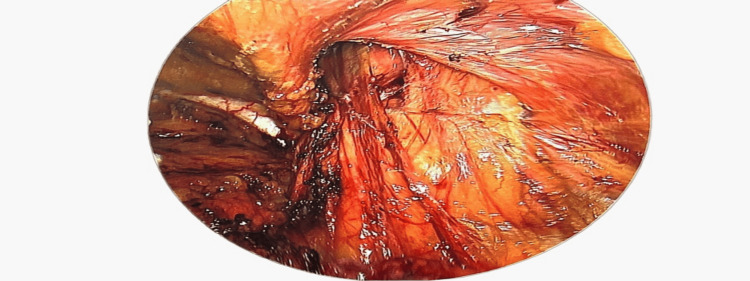
After dissection along the sac and isolating the cord contents.

**Figure 4:**
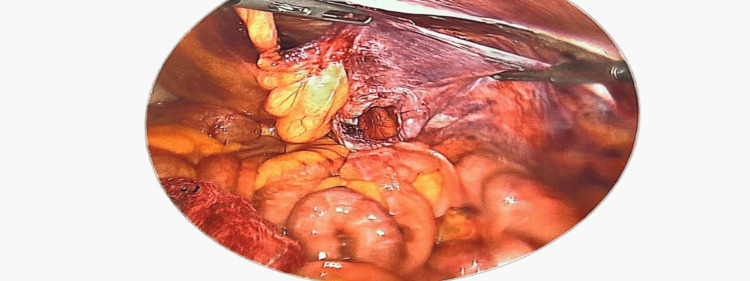
Creating a pre-peritoneal plane for placement of the mesh.

**Figure 5:**
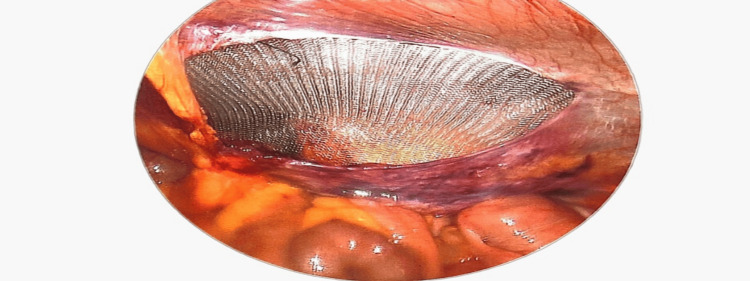
Placement of mesh with adequate cover in the pre-peritoneal plane.

**Figure 6:**
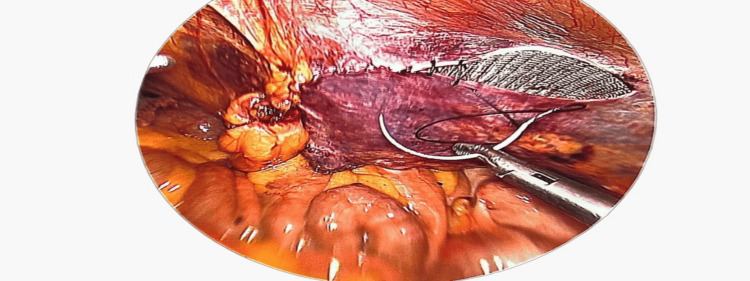
Closing the pre-peritoneal flap to avoid any contact of mesh with the intra-abdominal contents.

**Figure 7:**
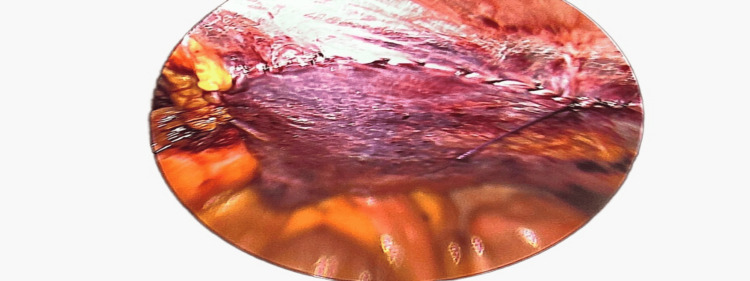
After complete closure of the pre-peritoneal plane/flap.

For concurrent Spigelian hernias, the peritoneal incision was extended laterally to include the defect, and the contents were reduced before placement of a preperitoneal Prolene mesh. In cases of concurrent umbilical hernias, an initial open approach was used to identify the linea alba defect, through which the 10 mm port was introduced. Defects smaller than 2 cm were closed primarily at the end of the procedure, while larger defects were repaired using mesh. (Figures 1-7)

Postoperative management

Most patients were managed as day-case admissions. Early mobilization was strongly encouraged. The postoperative pathway included regular analgesia for three days, use of thrombo-embolic deterrent (TED) stockings, and laxatives when required. Patients were advised to mobilize adequately. Follow-up was arranged as a face-to-face outpatient clinic review two to four weeks after surgery, with a telephone clinic follow-up in six months. 

## Results

Laparoscopic TAPP hernia surgeries were performed on 5,124 patients, comprising 4,252 (83%) males and 872 (17%) females. A wide range of body mass index values was included (BMI range 19-46) (Table 1). Of these patients, 1,333 had bilateral hernias, 249 had recurrent inguinal hernias, and 921 had femoral hernias. Additionally, other hernias repaired during the same operation included 189 paraumbilical hernias and 16 spigelian hernias. 

**Table 1:** Showing the different parameters in our study cohort. BMI: body mass index.

Parameters	Number
Gender	Male: 4,252 (83%); Female: 872 (17%)
BMI	Range 19-46 (Mean 31)
Age	18-91 years range

Operative outcomes

**Figure 8:**
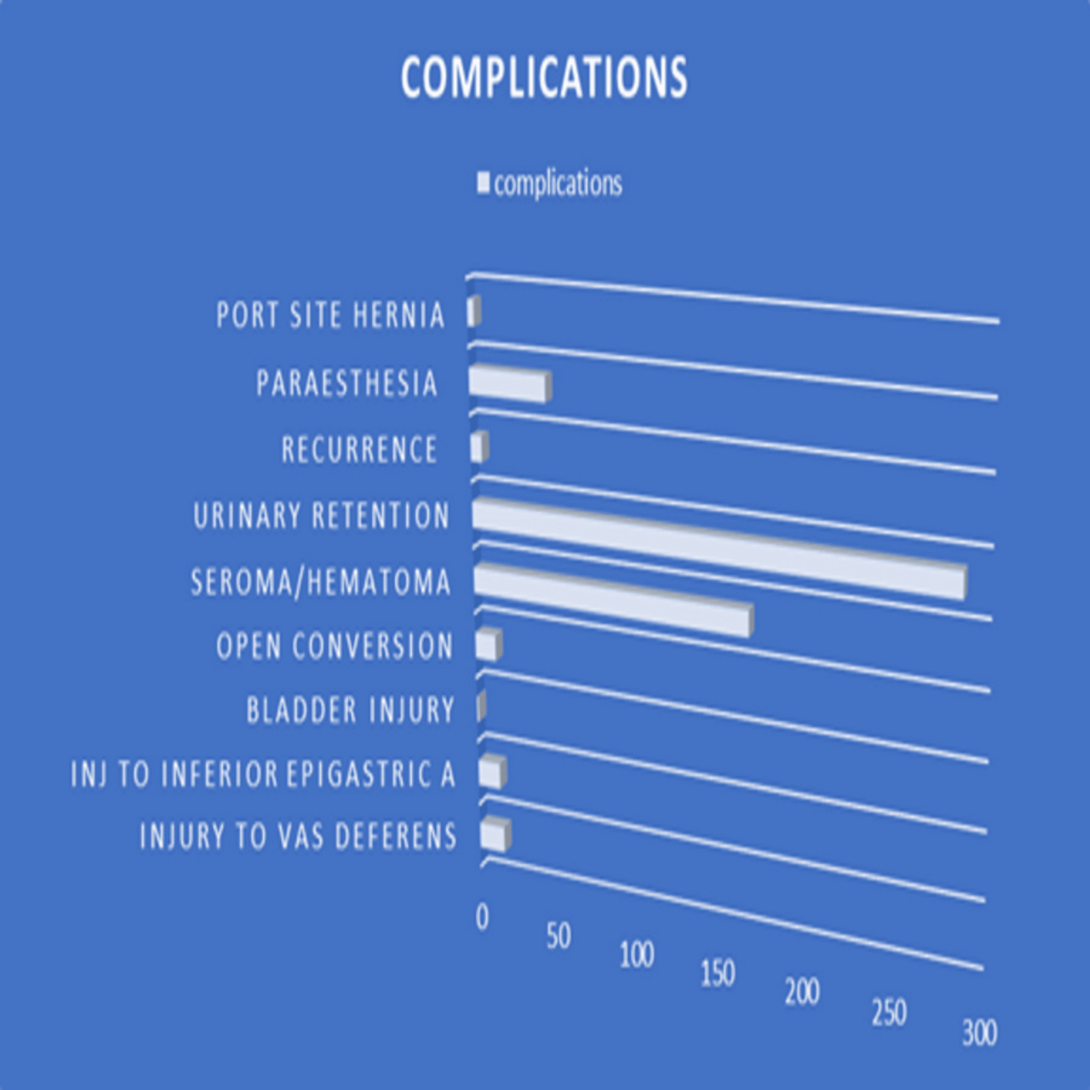
Showing complications in our cohort.

The majority of procedures were completed successfully using a laparoscopic approach. Conversion to open surgery was required in 13 patients, representing a very small proportion of the total cohort. Of these, eight conversions were due to extensive intra-abdominal adhesions, seven related to previous abdominal surgery, and one following pelvic radiotherapy. Five additional conversions were performed due to the irreducibility of the hernia contents despite careful laparoscopic traction (Figure 8). Large inguinoscrotal hernias, partially reducible hernias, and hernias associated with prior abdominal surgery were managed laparoscopically in most cases.

Postoperative outcomes and complications

Most patients were managed as day-case admissions, with early mobilization and standardized postoperative analgesia. Postoperative complications were infrequent. Seroma formation was observed predominantly in patients with large or wide-neck hernias and was managed conservatively, resolving without the need for intervention in the majority of cases.

No cases of port-site incisional hernia were observed following the adoption of the lateral 10 mm port placement technique. Urinary catheterization in selected patients over 60 years of age and those undergoing bilateral repair was not associated with prolonged urinary retention.

Recurrence and re-intervention

The recurrence rate in this series was low. Analysis of recurrence cases revealed that failures occurred at the edges of the mesh, rather than through the mesh itself. These findings emphasized the importance of wide pre-peritoneal dissection and adequate mesh overlap in preventing recurrence. Lessons learned from early recurrences informed refinements in operative technique, particularly regarding mesh positioning and fixation.

Laparoscopic management of obstructed and strangulated groin hernias was feasible in the majority of cases. Most hernias were reducible following induction of anesthesia or gentle laparoscopic traction. In cases where the hernia contents were viable after reduction, mesh placement was deferred, and elective repair was performed approximately four weeks later. When non-viable contents were encountered, resection was performed laparoscopically or via a limited midline incision as clinically indicated.

## Discussion

Laparoscopic repair of groin hernias has become the standard of care in many centres. In our experience, the TAPP approach offers significant advantages, including diagnostic access to the peritoneal cavity and excellent visualization of anatomy within a large operative field. Repair of a unilateral hernia does not compromise future dissection should a contralateral hernia develop. In addition, TAPP provides the opportunity for a general diagnostic laparoscopy. This technique is associated with favorable outcomes and typically has a relatively short learning curve [7]. The incidence of occult or incidental contralateral hernias detected during TAPP is approximately 25% [8].

Over time, our inclusion criteria have expanded to include all patients aged over 18 years. We have successfully operated on patients with a BMI of up to 46 and those with previous abdominal surgery, as adhesions can usually be safely dissected. The only contraindication to laparoscopic groin hernia repair in our practice is a history of laparoscopic radical prostatectomy, due to dense preperitoneal adhesions that significantly increase the risk of complications. These patients are therefore advised to undergo an open repair. Previous pelvic irradiation and prostatic interventions further compromise the operative field and increase the risk of iatrogenic injury [9].

A peritoneal incision is made above the upper margin of the hernia defect, allowing wide dissection from the midline to the anterior superior iliac spine. This facilitates adequate mesh overlap and enables identification of additional defects. Our standard approach is complete dissection and reduction of the hernia sac. However, in long inguinoscrotal sacs, circumcision of the sac may be required, leaving the distal portion within the scrotum. This technique minimises the risk of cord injury and postoperative scrotal haematoma. Clear identification of key anatomical landmarks, including the pubic bone, inferior epigastric vessels, vas deferens, spermatic cord, and round ligament in females, is essential.

Meticulous dissection with careful haemostasis reduces the risk of chronic seroma or pseudo-hydrocele formation [10]. Bittner et al. demonstrated minimal risk of orchitis or testicular injury following complete sac reduction [11]. The upper peritoneal flap is dissected to expose the superior edge of the defect and ensure a minimum mesh overlap of 3 cm. The lower flap is dissected to expose the psoas muscle while protecting the vas deferens and spermatic cord. In female patients, division of the round ligament facilitates wide dissection of the lower flap. Wide dissection is emphasized to ensure complete coverage of all potential weak areas, as hernia recurrence typically occurs at the edges of the mesh rather than through it. This principle is supported by international guidelines [12].

While wide peritoneal flap dissection may reduce recurrence rates, it can prolong operative time and increase the risk of iatrogenic injury, particularly to neural structures and the vas deferens [13, 14]. Excessive dissection may also predispose to seroma formation or peritoneal tears [15].

Hernia contents are reduced using gentle traction, ensuring that preperitoneal fat is fully reduced and differentiated from a true cord lipoma. Care is taken to avoid injury to the spermatic cord. In femoral hernias, the lateral division of the lacunar ligament may facilitate safe reduction, while vigilance is required to avoid injury to femoral, iliac, or obturator vessels. Transitioning from a three-port to a four-port technique, with lateral placement of a 10 mm port near the anterior superior iliac spine, has eliminated port-site incisional hernias in our series.

A 10 × 15 cm polypropylene (Prolene) mesh is used in all cases. Depending on the extent of dissection, two or three meshes may be required to ensure adequate coverage from the midline to the anterior superior iliac spine. Meshes are shaped appropriately and fixed with a single suture to prevent migration, with absorbable tackers used selectively for wide defects. Meticulous closure of the peritoneal defect is critical to prevent adhesions, bowel obstruction, or mesh erosion [16]. Closure is achieved using running sutures, tackers, or glue [14]. In large inguinoscrotal hernias, circumcision of the sac is often required, with particular care taken near the inferior epigastric vessels, which may occasionally need to be divided to facilitate safe reduction.

Peritoneal flaps are closed with continuous 2/0 Vicryl sutures, and any residual defects are closed individually to prevent internal herniation. Recurrence in our series has been low and has informed our emphasis on wide, flat mesh placement with adequate overlap in all directions.

Patients presenting with chronic groin pain without clinical or ultrasonographic evidence of hernia are challenging to manage. These patients undergo dynamic groin ultrasound, pelvic MRI, and orthopaedic assessment. If no diagnosis is established, diagnostic laparoscopy is performed, as small occult hernias containing preperitoneal fat may be present. 

Although initially considered a contraindication, laparoscopic management of obstructed groin hernias is now routinely attempted in our practice. Most can be safely reduced using gentle circumferential traction. The peritoneal neck is closed, and elective mesh repair is performed four weeks later to minimise infection risk. Non-viable contents are resected laparoscopically or via a small midline incision as required.

For wide defects, we have observed that overlapping meshes may reduce seroma formation, although this remains anecdotal. Injuries to the inferior epigastric vessels or vas deferens are managed by securing both ends with clips or sutures to prevent bleeding and reduce postoperative complications, including ejaculatory pain.

An important limitation of this study is that it reflects the evolving practice of a single surgeon, which may limit its generalisability. The technique is therefore presented as an experiential insight rather than definitive evidence. Additionally, follow-up within our cohort was limited, which may affect outcome assessment; however, to the best of our ability, we traced available follow-up data to evaluate outcomes.

## Conclusions

Our experience indicates that TAPP hernia repair can be performed safely and effectively, with low rates of intraoperative and postoperative complications. In experienced hands, TAPP represents a dependable minimally invasive option for inguinal hernia repair. In our experience, TAPP repair remains a robust approach for inguinal hernia surgery, combining excellent exposure with the benefits of minimally invasive surgery. With meticulous pre-peritoneal dissection, adequate mesh overlap, secure fixation, and careful peritoneal closure, low complication and recurrence rates can be achieved across a broad patient population, irrespective of age, body mass index, or previous abdominal surgery. When performed by experienced surgeons, TAPP repair can offer durable outcomes with low morbidity and a strong potential for day-case surgery, supporting its ongoing role as a key technique in contemporary hernia practice. Surgeons need to choose surgical techniques with which they are experienced and that offer the greatest benefit to their patients. Despite recent advances in robotic surgery, laparoscopic techniques continue to hold significant value in the management of hernias. It will be of interest to determine whether the TAPP approach becomes the standard technique for the management of emergency hernia repair in the future.
